# High-entropy alloy strengthened by *in situ* formation of entropy-stabilized nano-dispersoids

**DOI:** 10.1038/s41598-018-32552-6

**Published:** 2018-09-20

**Authors:** Bharat Gwalani, Rizaldy M. Pohan, Junho Lee, Bin Lee, Rajarshi Banerjee, Ho Jin Ryu, Soon Hyung Hong

**Affiliations:** 1 Department of Materials Science and Engineering, University of North Texas, 1155 Union Circle, Denton, TX 76203 USA; 2 Department of Materials Science and Engineering, Korea Advanced Institute of Science and Technology, 291 Daehak-ro, Yuseong-gu, Daejeon, 34141 Republic of Korea; 3 Korea Institute of Rare Metals, Gaetbeol-ro 12, Incheon, Republic of Korea; 4 Department of Nuclear and Quantum Engineering, Korea Advanced Institute of Science and Technology, 291 Daehak-ro, Yuseong-gu, Daejeon, 34141 Republic of Korea

## Abstract

A significant increase in compressive yield strength of the Al_0.3_CoCrFeMnNi high-entropy alloy (HEA) from 979 MPa to 1759 MPa was observed upon the introduction of 3 vol.% Y_2_O_3_. The HEAs were processed using spark plasma sintering of mechanically alloyed powders. Transmission electron microscopy and atom probe tomography confirmed the presence of compositionally complex nano-dispersoids in the Y_2_O_3_-added HEA. The significant increase in strength can be attributed to the nano-dispersoid strengthening coupled with grain refinement. Therefore, the *in-situ* formation of the compositionally complex nanoscale dispersoids during the alloy processing could be a novel approach to create entropy-stabilized oxide particles in strengthening of HEAs.

## Introduction

High-entropy alloys (HEAs) are a new class of metallic alloys, defined by Yeh *et al*.^1^ as an alloy system consisting of five or more metallic elements with concentrations in the range of 5–35 at%. As their name suggests, HEAs exhibit a high configurational entropy, which favors the formation of a solid solution instead of intermetallic compounds. They attract significant research interests owing to their high strength, thermal stability, wear resistance, corrosion resistance, etc.

Amongst the myriad of HEA systems, CoCrFeMnNi is one of the most intensively investigated alloys owing to its attractive properties including cryogenic mechanical properties^2^, thermodynamic stability^3^, and malleability. Its microstructure consists of a single face-centered-cubic (*fcc*) solid solution, initially revealed by Cantor *et al*.^4^.

Although it exhibits various desirable properties, the mechanical strength of CoCrFeMnNi is very low^5^. CoCrFeMnNi HEA is commonly synthesized using melting and casting processes. However, ingot metallurgy tends to create coarse grains with heterogeneous dendritic structures during cooling^6^. In order to improve its mechanical properties, additional thermomechanical methods have to be employed, such as rolling, high-pressure torsion^3^, and swaging^7^, after arc melting.

An alternative method to synthesize HEAs is powder metallurgy processing including mechanical alloying (MA) and spark plasma sintering (SPS). A target material, in the form of powder, is subjected to a repetitive cycle of cold welding, fracturing, and rewelding using high-energy ball milling to achieve solid-state alloying. The alloyed powders are then subjected to compaction and sintering. Using the powder metallurgy process, nanograins can be obtained, which improve the mechanical properties of the HEA^8^.

The addition of aluminum to CoCrFeMnNi can enhance its mechanical properties through the increase of the lattice distortion. He *et al*.^9^ reported that the addition of Al not only decreased the density of the alloy but also increased the yield strength through solid solution strengthening and formation of a harder body-centered cubic (*bcc*) phase^10,11^ with the further addition of aluminum.

Another strengthening method is oxide dispersion strengthening (ODS), based on oxides such as Al_2_O_3_, TiO_2_, ZrO_2_, and Y_2_O_3_, which restrict dislocation motion by dispersion strengthening and restrain grains’ growth owing to the grain-pinning effect^12^. Owing to its high hardness (~1,020 Hv) and thermal stability^13^, Y_2_O_3_ is commonly used as a dispersoid for ODS alloys. ODS effectively strengthened the CoCrFeMnNi HEA from 1 GPa to 1.2 GPa at room temperature, and from 400 MPa to 800 MPa at 800 °C^14^. Even though Al-containing HEAs have been extensively investigated owing to the promising strength-ductility combinations^1,9,15^, there are no reports on ODS of Al-containing CoCrFeMnNi HEAs. This study focuses on the effect of introducing Y_2_O_3_ in the Al_0.3_CoCrFeMnNi HEA, fabricated using MA and spark plasma sintering. The *in-situ* reaction of Y_2_O_3_ with the other substitutional elements (from the solid-solution HEA matrix) leads to a complex dispersoid. This is the first report that employs atom probe tomography (APT) to accurately measure the composition and morphology of the nanoscale oxide dispersoids.

## Results and Discussion

### XRD analyses during MA and after SPS

**Figure 1:**
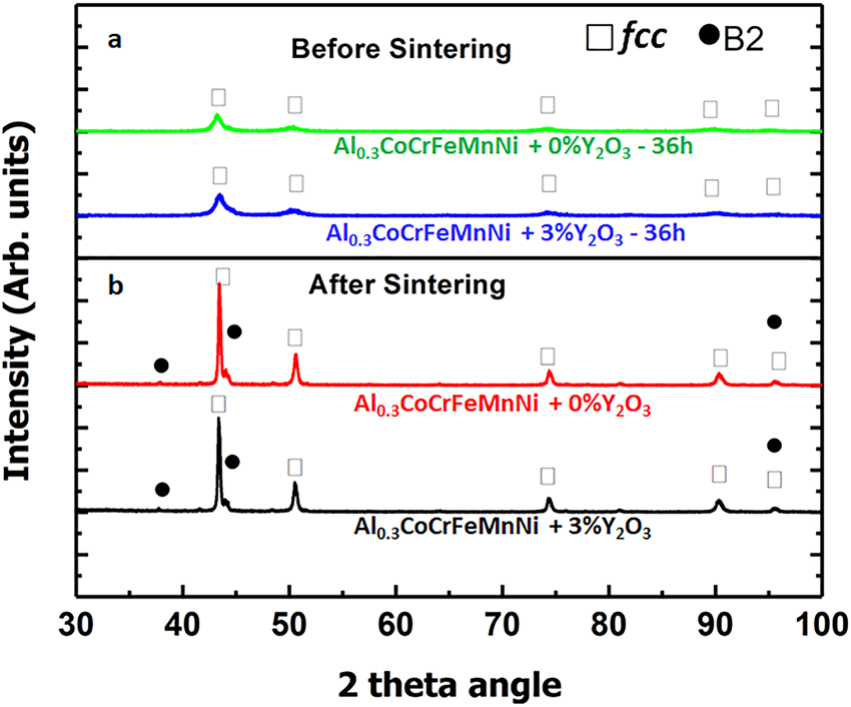
X-ray Diffraction: (**a**) XRD peaks of Al_0.3_CoCrFeMnNi without Y_2_O_3_ and with 3% at. Y_2_O_3_ after 36 hours of milling. (**b**) XRD peaks of sintered Al_0.3_CoCrFeMnNi without Y_2_O_3_ and with 3% at. Y_2_O_3_ after milling.

Figure 1(a) shows the XRD results after the milling of the Al_0.3_CoCrFeMnNi powders. After 36 h of milling, only *fcc* peaks were observed for both samples, alloy powders without Y_2_O_3_ (0% Y_2_O_3_) and with 3% Y_2_O_3_. The milling conditions optimized for Al_0.3_CoCrFeMnNi high entropy alloys were used in this study. The details of the milling conditions and the microstructural evolution of the mechanically alloyed powder were described in our previous paper by the authors on Al_0.3_CoCrFeMnNi^16^. Figure 1 shows that the diffraction peaks of Al_0.3_CoCrFeMnNi and ODS HEA are almost similar because the addition of Y_2_O_3_ did not change the milling process significantly The *fcc* peaks had a low intensity and were relatively broadened, indicating a decrease in the crystallite size, according to the Scherrer’s formula.

The mechanical milling was followed by a sintering process. The optimal sintering temperature of 900 °C was optimized by measuring relative densities of Al_0.3_CoCrFeMnNi (>99%, indicating that a full densification) and by performing a post hardness test and grain-size measurements in the previous study^16^. After sintering, the XRD peaks exhibited a high intensity and were narrower, compared with those before the sintering, as shown in Fig. 1(b). Both Al_0.3_CoCrFeMnNi (without Y_2_O_3_) and Al_0.3_CoCrFeMnNi + 3% Y_2_O_3_ exhibited a combination of *fcc* (major phase) and B2 peaks (minor phase). The B2 crystal structure is often adopted by AB-type compounds and has been reported in many Al containing HEAs^1,11,16^. It has been previously reported that the volume fraction of the B2 phase in Al_0.3_CoCrFeMnNi is approximately 6 vol%^16^. The B2 phase has a space group of Pm3m, which is a primitive cubic structure. The chemical ordering within the B2 phase is based on the ordering of a body-centered cubic structure with different atomic species occupying the corners of the cube versus the body center of the cube. B2 alloys often exist over a range of compositions on either side of the stoichiometric composition. Deviations from the stoichiometric composition are accommodated by constitutional defects, i.e. either by vacancies on the deficient element’s sublattice sites or by antisite atoms of the excess element^17^. In some compounds, e.g. aluminum-rich NiAl, the vacancies may be ordered^18^. The mechanical properties of B2 can be profoundly influenced by the composition of the intermetallic phase^17^ and hence this phase in compositionally complex alloys like HEAs can be tuned over a wide range of compositions for tuning the mechanical properties.

### Microstructural characterization of the constituent phases

**Figure 2:**
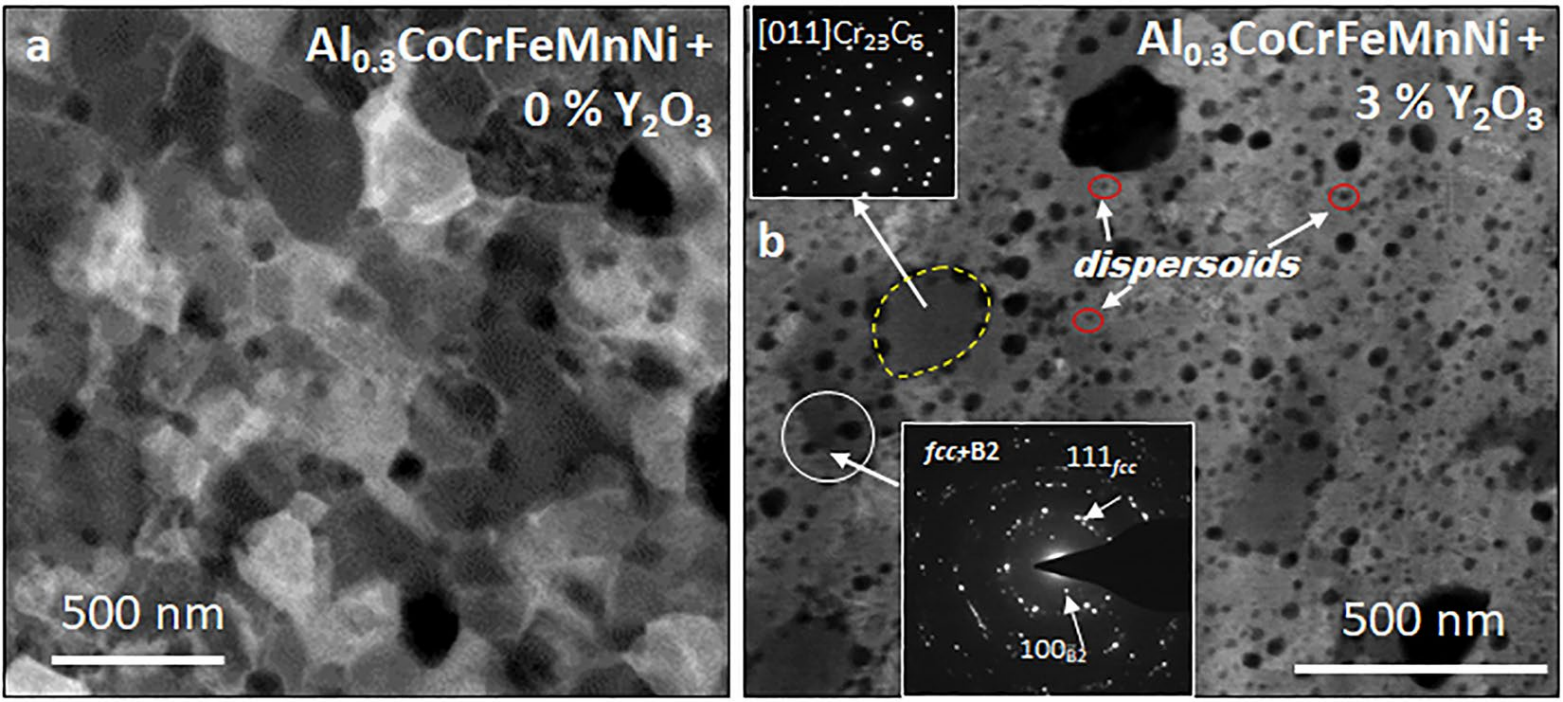
Transmission Electron Microscopy: (**a**) STEM image from Al_0.3_CoCrFeMnNi + 0% Y_2_O_3_ showing the nano-grains formed by the SPS process. (**b**) STEM image from Al_0.3_CoCrFeMnNi + 3% Y_2_O_3_ shows the nano-grains along with the dark contrast dispersoids. Inset on top shows the SADP from Cr-carbide, and that on the bottom shows SADP from *fcc* + B2 phases.

Detailed microstructural studies of the Al_0.3_CoCrFeMnNi (without Y_2_O_3_) sample showed the presence of *fcc*, B2, and chromium carbide phases^16^. Carbon measurements throughout the process showed that the alloy absorbed ~0.25 at% C after the mechanical milling, which reached the value of 0.4 at% C after the sintering in graphite dies; metal-carbides are often observed in SPS-processed alloys^16,19,20^. A scanning TEM (STEM) image of Al_0.3_CoCrFeMnNi (without Y_2_O_3_) is shown in Fig. 2(a), which shows the fully recrystallized microstructure consisting of nanograins. Some of the grains exhibit a darker contrast in Fig. 2a. This darker contrast can be attributed to carbides (Cr and C rich), and the B2 particles (Al, Mn and Ni rich) which are composed of relatively lighter elements and consequently appear darker in contrast in HAADF STEM images. However, some of the fcc grains may also appear dark due to diffraction contrast effects and strain differences.

The STEM image and selected area diffraction patterns (SADPs) of the phases observed in Al_0.3_CoCrFeMnNi + 3% Y_2_O_3_ are shown in Fig. 2(b) (and insets). The inset in the top-left part shows an SADP of the M_23_C_6_ carbide phase along the [011] zone axis (outlined by the dotted yellow shape). The inset in the bottom-middle part shows an SADP of the *fcc* nanograins and B2 particles. These phases are formed during the sintering and do not contain Y_2_O_3_, as reported in our previous study^16^. Apart from the major sintered phase, small dark-contrast spherical features dispersed in the microstructure of Al_0.3_CoCrFeMnNi + 3% Y_2_O_3_ were observed. These features were absent in the alloy without Y_2_O_3_, suggesting that they may be the dispersoids formed upon the addition of 3% Y_2_O_3_. However, it was challenging to precisely characterize the dispersoids using TEM; APT was employed for this purpose.

### Compositional studies using APT

**Figure 3:**
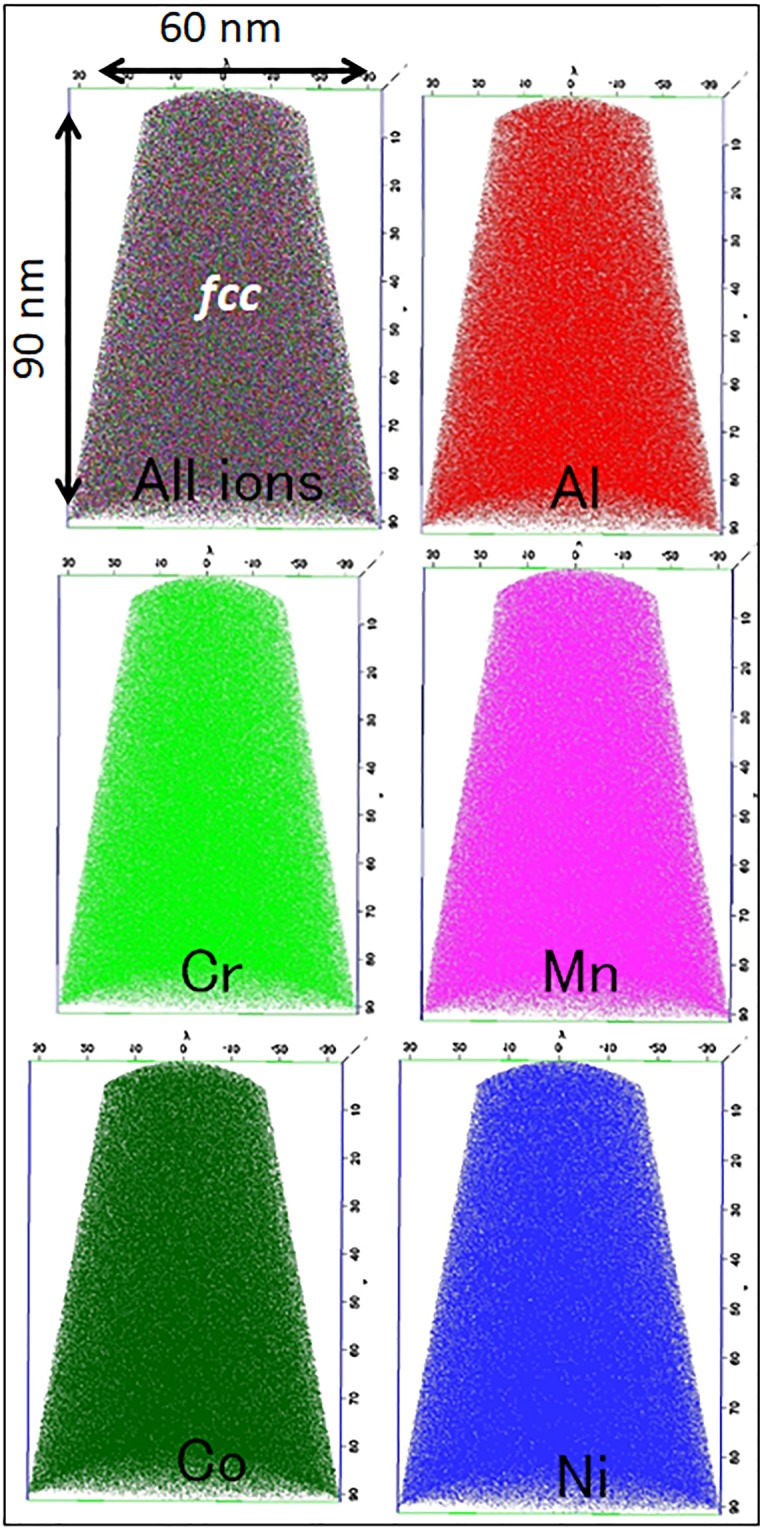
Atom Probe Tomography: 3-D reconstruction of APT data from Al_0.3_CoCrFeMnNi + 0% Y_2_O_3_ showing a homogeneous distribution of all elements.

**Figure 4:**
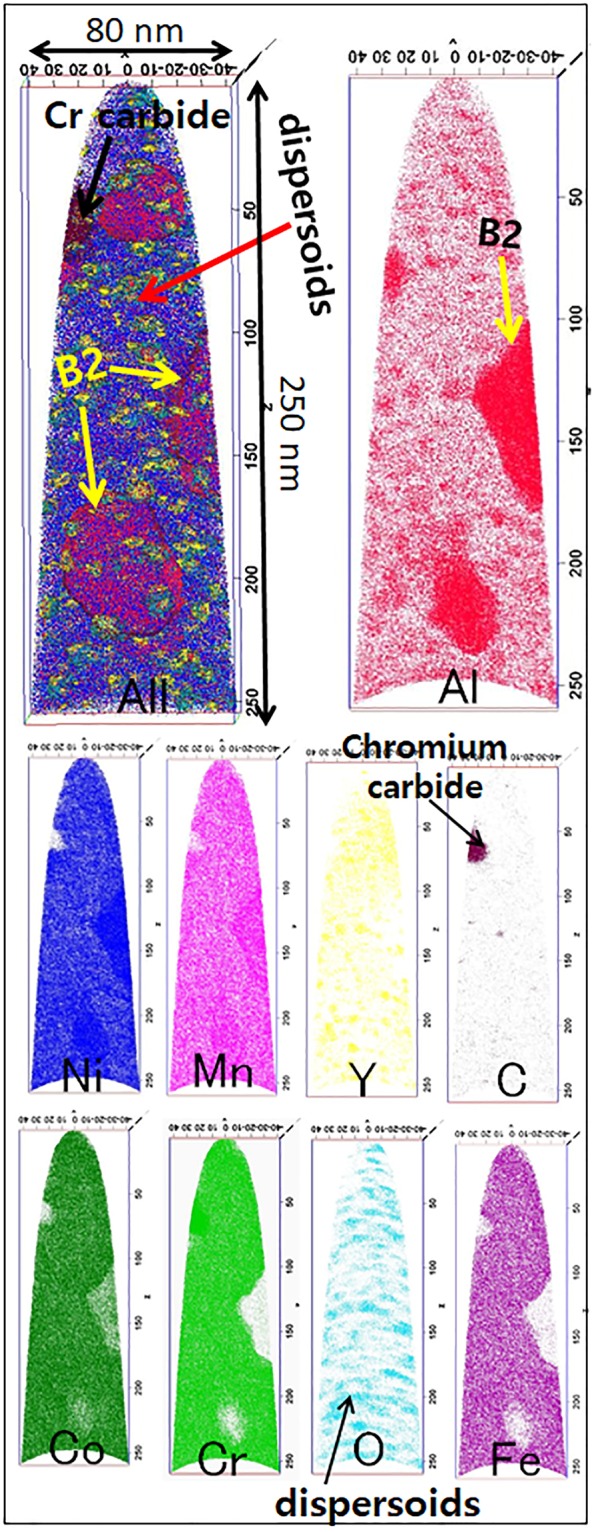
Atom Probe Tomography: 3-D reconstruction of APT data from Al_0.3_CoCrFeMnNi + 3% Y_2_O_3_ showing the presence of various features as labeled in the figure. Top left reconstruction shows an all-ions map followed by the reconstructions showing individual ion maps for various elements. Fine scale inhomogeneity within the matrix clearly seen in Al, Y and O maps correspond to the dispersoids.

APT results for the 0%-Y_2_O_3_ and 3%-Y_2_O_3_ alloys are shown in Figs 3 and 4. Raw ion maps (Al (red), Cr (light green), Mn (magenta), Co (dark green), and Ni (blue)) from the *fcc* “matrix region” of the 0%-Y_2_O_3_ sample are shown in Fig. 3. The performed reconstruction reveals very homogeneous distributions of each of the chemical elements.

Ion maps of the 3%-Y_2_O_3_ alloy are shown in Fig. 4. Apart from the *fcc* matrix, the reconstruction reveals various microstructural features. In contrast to the 0%-Y_2_O_3_ sample, the *fcc* matrix in the 3%-Y_2_O_3_ alloy consists of fine-scale inhomogeneities, which are the distributed dispersoids, observed in the Al, Y, and O ion maps. Larger Al–Ni–Mn-rich particles are B2 precipitates formed in the alloy. In addition, a chromium–carbon-rich region is observed in the top-left part of the APT tip, which is the Cr-rich carbide (M_23_C_6_) formed during the SPS processing of the powders. Increases of the Al, Y, and O intensities within these regions can be clearly observed by visual inspection; the exact compositions of these phases are provided in Table 1. It is worth noting that Y_2_O_3_ can react with Al, forming a Y–Al–O complex oxide, *in-situ*, during the sintering of the powders. Amongst the possible oxides, yttria alumina monoclinic, yttria alumina perovskite, and yttria alumina garnet, have been reported^21,22^. However, the high contents of the other elements, Co, Cr, Fe, Mn, and Ni, suggest that owing to the high entropy state of the system, a Y–Al–Mn–Ni–Cr–Co–O complex oxide is formed through a surface reaction with the matrix^23–25^.

**Table 1:** Compositions of various phases by atom probe tomography of Al_0.3_CoCrFeMnNi + 3% Y_2_O_3_.

Phase	Compositions [at%]							C	O
	Al	Co	Cr	Fe	Mn	Ni	Y		
*fcc*	5.7 ± 0.09	18.3 ± 0.03	15.7 ± 0.05	19.3 ± 0.4	18.4 ± 0.03	21.2 ± 0.08	1.04 ± 0.02	0.1 ± 0.1	1.01 ± 0.02
B2	19.8 ± 0.04	10 ± 0.03	0.7 ± 0.1	3.9 ± 0.04	24.9 ± 0.01	38.2 ± 0.01	2.3 ± 0.07	0	1.9 ± 0.1
Cr-carbide	0	4.5 ± 0.09	59.3 ± 0.09	9.8 ± 0.08	8.8 ± 0.03	4.3 ± 0.05	0	12.7 ± 0.02	0
Dispersoid	8.7 ± 0.07	12.1 ± 0.04	9.2 ± 0.05	13 ± 0.07	16.1 ± 0.04	20.3 ± 0.03	9.9 ± 0.04	0	9.7 ± 0.03

In addition, studies on ODS of Al- and Ti-containing alloys demonstrated the formation of similar non-stoichiometric sub-oxide nanoprecipitates^25–27^. Ceri *et al*.^25^ and Lindau *et al*.^27^ suggested that, after MA, Y_2_O_3_ might not dissolve completely, and that the cores of the nanofeatures observed after sintering can consist of undissolved Y_2_O_3_. The cores can act as nuclei for other elements to segregate at the interface, which lowers the interface energy. London *et al*. showed that Cr could form a shell around oxide particles, leading to particles with a very small size and increased number density^28^. The composition of the oxide particles was determined to be ~9% Al, 12% Co, 9% Cr, 13% Cr, 16% Fe, 20% Ni, 10% Y and 10% O (all in at%). The high compositional complexity suggests substantially higher configurational entropy as compared to conventional Y_2_O_3_ particles in solute lean alloys. The development of entropically stabilized oxides (or high entropy oxides) has been an active area of interest in recent times^29–34^. Rost *et al*.^29^ published on the existence of a new class of entropy stabilized mixed oxides with high configurational entropy. The authors of^29^ also presented a hypothesis suggesting that entropy stabilization is particularly effective in a compound with ionic character. Lei *et al*.^30^ developed ultrastable metal oxide nanotube arrays consisting of multiple oxide constituents by anodic oxidation of high-entropy alloy precursors. This has implications in the field of catalysis and energy storage.

The volume fraction of dispersoids (determined by APT) in the 3 vol.%Y_2_O_3_ reinforced alloy was determined to be 0.033. The radius of dispersoid particles was also determined from the APT analysis using iso-concentration surfaces to encapsulate the Al-rich dispersoids. The average radius of dispersoids was 0.95 nm in case of the 3 vol.% Y_2_O_3_ reinforced alloy. Further studies are ongoing to investigate the effect of the Y_2_O_3_ content on the composition and size of the complex oxide dispersoids formed in the alloy.

**Figure 5:**
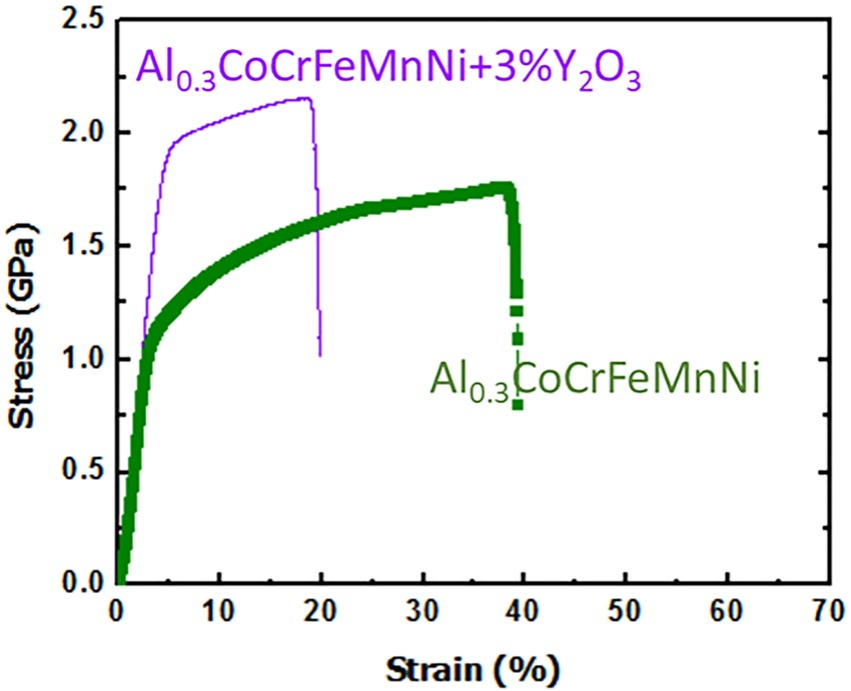
Performance under mechanical loading. (**a**) Compressive stress-strain curves of Al_0.3_CoCrFeMnNi 0% Y_2_O_3_ and ODS Al_0.3_CoCrFeMnNi with 3% Y_2_O_3_.

Compressive stress–strain curves for the Al_0.3_CoCrFeMnNi alloys without Y_2_O_3_ and with 3% Y_2_O_3_ at ambient temperature are shown in Fig. 5. At 0% Y_2_O_3_, Al_0.3_CoCrFeMnNi exhibits a yield strength of 979 MPa with a strain of 39.3%; at 3% Y_2_O_3_, the yield strength increased to 1,759 MPa (an increase of ~80%), while the strain decreased to 19.8%. This indicates that the Y_2_O_3_ dispersoids act as a barrier to dislocation motion, enhancing the mechanical properties, still maintaining a reasonable amount of ductility.

Considering the sample processing parameters and apparatus used for processing the base alloy (0% Y_2_O_3_) and the 3% Y_2_O_3_ reinforced alloy were same, the Hall-Petch strengthening due to grain size effect, composite strengthening due to intermetallic B2 and carbide particles and solid solution strengthening can be assumed similar in both alloys. Hence, the yield strength of the base, which is ~980 MPa if assumed to be due to the grain size and solid solution strengthening, the strength increment on reinforcing the alloy with 3% Y_2_O_3_ can be estimated to be ~780 MPa. Theoretically, the dispersion strengthening can be modeled using the dispersed-barrier hardening model^35^. This model is based on the intersection of the obstacles with the dislocation glide plane^36^ using geometrical estimations:

1 $${\rm{\Delta }}{\rm{\sigma }}=0.8\,{\rm{M}}{\rm{\alpha }}\,({\rm{r}}){\rm{Gb}}/\lambda $$

where α(r) is the obstacle strength-coefficient and it represents the strength of the obstacles to impede dislocation motion. Alinger^37^ proposed a function of α(r),

2 $${\rm{\alpha }}({\rm{r}})=-\,0.017+0.374\,\mathrm{log}(\frac{{\rm{r}}}{2{\rm{b}}})$$

in case of an impenetrable obstacle, α(r) = 1 and Orowan by-pass mechanism can be used. In the current work, α(r) is ~0.08 where a range of α(r) values approximately lying between 0.05 and 0.30 clearly indicates that the fine scale nanodispersoids are relatively soft obstacles, and can be sheared by dislocations^38^.

G, b (0.255 nm for CoCrFeNiMn)^39^, f and r denote the shear modulus, the Burgers vector and the dispersoid size, respectively. *λ* is the average planar center-to-center distance between nano-dispersoids and is given by:

3 $${\rm{\lambda }}=2\sqrt{\frac{2}{3}}\,{\rm{r}}(\sqrt{\frac{\pi }{4f}}-1)$$

In current work it is noted that the direct-strengthening calculated from the dispersed-barrier hardening model is about one-third of the Orowan by-pass model. Siska *et al*. in their assessment of strengthening mechanisms arising from different oxide particles in 9Cr ODS steel showed that the dispersed-barrier hardening model works better for Y_2_O_3_ dispersoids^40^.

The average radius of dispersoids was 0.95 nm in case of the 3 vol.% Y_2_O_3_ reinforced alloy. This APT data reveals dispersion strengthening (σ, according to Eq. (1)) in 3 vol.% Y_2_O_3_ reinforced Al_0.3_CoCrFeMnNi alloy was ~695 MPa in good agreement with the experimentally estimated strength estimate of ~780 MPa. This establishes that the dispersion strengthening mechanism plays an important role in imparting high compressive strength in such ODS HEAs. The difference in the experimental value and the calculation could be explained based on the non-additive nature of the various mechanisms. The strengthening contributions due to Hall-Petch, solid solution strengthening and dispersion strengthening can influence each other and the dislocation density of the matrix. This will also change the dislocation strengthening of the base matrix.

**Figure 6:**
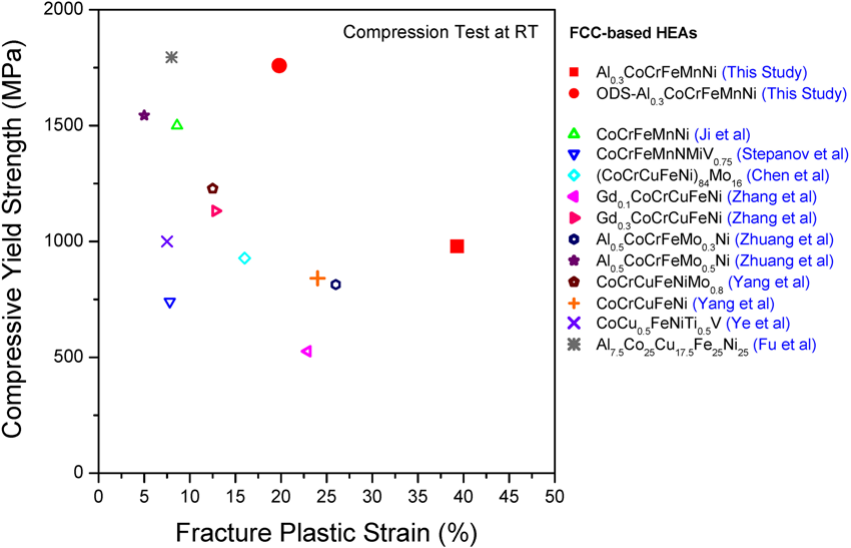
The plot of compressive yield strength-fracture strain combinations from various fcc-containing high strength HEAs including the current alloys, showing great advantage of the current HEAs.

Furthermore, a comparison with other *fcc*-based HEAs^41–49^ is summarized in Fig. 6 where the compressive YS vs fracture strain is presented. The analyzed 3%-Y_2_O_3_ HEA is located in the upper-right part of the diagram, which indicates that it outperforms most of the reported HEAs. It seems that the current alloy has the most desirable combination of strength and ductility among *fcc*-based HEAs materials. For a study of the detailed deformation mechanisms of dispersion-strengthened HEAs, tensile tests, creep and fatigue tests should be conducted in the future.

## Conclusion

In this study, Al_0.3_CoCrFeMnNi + 3 vol.% Y_2_O_3_ HEAs were prepared by planetary ball milling for 36 h followed by spark plasma sintering at 900 °C under a pressure of 50 MPa for 10 min. The SPS yielded a nano-grained microstructure consisting of *fcc*, B2, and M_23_C_6_ carbide phases. Along with these phases, the alloy exhibited an even finer-scale complex-oxide dispersed phase, formed through an *in-situ* reaction during the sintering, whose morphology was investigated using APT. The dispersoids led to an increase in the yield strength and a decrease in ductility. The large increase in the yield strength could be attributed to the dispersion strengthening, indicating the effectiveness of the employed strengthening method. This study presented a novel methodology for the formation of complex oxide dispersoids in compositionally complex HEAs, which can also be applied to other alloys.

## Methods

High-purity powders of Al, Co, Mn, Ni (particle size <15 μm), Cr (particle size <45 μm) (Kojundo Co, Ltd.), Fe (particle size <25 μm), and Y_2_O_3_ (particle size <10 μm) (Sigma Aldrich) were used to prepare Al_0.3_CoCrFeMnNi (expressed in molar ratio) with 3 vol.% Y_2_O_3_ by planetary ball milling for 36 h. Stainless-steel vials and stainless-steel balls with a diameter of 1.1 cm were employed, using a ball-to-powder mass ratio of 15:1 in an argon atmosphere including n-heptane as a process control agent (PCA) to reduce the cold welding of the powder. The as-milled powders were then consolidated using spark plasma sintering (Dr. Sinter Lab. SPS-515S) at 900 °C in a vacuum atmosphere (1.5 × 10^−5^ Bar) for 10 min under a uniaxial pressure of 50 MPa, at a heating rate of 100 °C/min.

The crystal structures of the milled powders and sintered alloys were investigated using an X-ray diffractometer (XRD, Rigaku D/Max-2500) with Cu-*K*_α_ radiation. Their microstructures were observed using transmission electron microscopy (TEM) (Tecnai G2 F30 S-Twin) and APT (Cameca 3000X HR local electrode atom probe (LEAP)). Thin TEM lamellae and tapered APT needles were produced from site-specific regions of interest, for TEM and APT analyses, respectively. The lamellae were prepared by focused ion beam (FIB) milling using an FEI Nova 200 NanoLab Dual Beam system equipped with a Ga+ ion source and Pt gas injection system. The densities of the sintered alloys were measured using the Archimedes’ principle. The compressive properties of the specimens (cylindrical shape, with a diameter of 3 mm and height of 6 mm) were investigated using an INSTRON 5583 system at a crosshead speed of 0.2 mm/min.

### Impact Statement

An unprecedented yield strength of Al_0.3_CoCrFeMnNi up to 1759 MPa was achieved by the *in-situ* formation of the compositionally complex nanoscale dispersoids in the high entropy alloy.

## Data Availability

The data that support the findings of this study are available within the paper. Any further information or clarification is available from the corresponding author upon reasonable request.
